# Can Virtual Assistants Perform Cognitive Assessment in Older Adults? A Review

**DOI:** 10.3390/medicina57121310

**Published:** 2021-11-29

**Authors:** Carmen Moret-Tatay, Isabel Iborra-Marmolejo, María José Jorques-Infante, José Vicente Esteve-Rodrigo, Carla H. A. Schwanke, Tatiana Q. Irigaray

**Affiliations:** 1 Faculty of Psychology, Universidad Católica de Valencia San Vicente Mártir, Burjassot, 46100 Valencia, Spain; isabel.iborra@ucv.es (I.I.-M.); mariajose.jorques@ucv.es (M.J.J.-I.); jv.esteve@ucv.es (J.V.E.-R.); 2 Dipartimento di Neuroscienze, Salute Mentale e Organi di Senso (NESMOS), Sapienza University of Rome, 00185 Rome, Italy; 3 Graduate Program in Biomedical Gerontology, School of Medicine, Pontifícia Universidade Católica do Rio Grande do Sul (PUCRS), Porto Alegre 90619-900, Brazil; schwanke@pucrs.br; 4 Institute of Geriatrics and Gerontology, Pontifícia Universidade Católica do Rio Grande do Sul (PUCRS), Porto Alegre 90619-900, Brazil; 5 Programa de Pós Graduação em Paicologia, Pontifícia Universidade Católica do Rio Grande do Sul, Porto Alegre 90619-900, Brazil; tatiana.irigaray@pucrs.br

**Keywords:** cognitive impairment, digitalization, virtual assistants, older adults

## Abstract

Community-dwelling older adults have raised the scientific community’s interest during the COVID-19 era as their chronic conditions might be aggravated by the consequences of confinement. Digital devices in this field to monitor cognitive impairment are an emerging reality of an innovative nature. However, some groups may not have benefited from these developments as much as, for example, younger people. The aim of this manuscript is to carry out a review on the development of digital devices, and specifically virtual assistants, for the detection of cognitive impairment in older adults. After a screening process, eight studies were found under the given criteria, and this number was even smaller for those using virtual assistants. Given the opportunities offered by virtual assistants through techniques such as natural language processing, it seems imperative to take advantage of this opportunity for groups such as older adults.

## 1. Introduction

Currently, society is experiencing the confluence of two immense revolutions within a health crisis due to the COVID-19 pandemic: (i) while biotechnology is unlocking the mysteries of the human body, particularly in the field of neuroscience, (ii) info-technology is providing us with unprecedented data processing power, giving rise to big data algorithms that will lead to a better understanding of human functioning, for most better than humans themselves. Thanks to the advance of digital technology, an opportunity emerges underlying these revolutions, including research trends related to virtual assistants. It should be noted that this technology can reduce the impact of most of the restrictions carried out to curb the virus spread thanks to digitalization. Had a similar situation arisen less than a decade ago, the impact would surely have been much greater. Even if different side effects have been described due to the increase of digitalization during lockdown, the preservation of most basic human rights, such as education or telehealth, have been possible through it. Information and communication technologies (ICT), as well as other communication systems based on artificial intelligence (AI), can collect and compare data of interest for healthcare, particularly for psychiatric approaches related to major mental health pathologies such as depressive disorder, bipolar disorder, schizophrenia, attention deficit hyperactivity disorder, or anxiety disorders, among others [1]. Virtual assistants based on AI are of interest in this front, as its potential in the health care systems has begun to improve the early detection and health care of mental illness, considering these systems as an aid and a complement to traditional therapies.

Community-dwelling older adults are considered one of the most vulnerable profiles during the COVID-19 era, and their chronic conditions might be aggravated by the consequences of lockdown. Not surprisingly, social-distancing measures to contain the COVID-19 outbreak would increase loneliness, which is also a well-known risk factor for poor physical and mental health [2]. Furthermore, this problem is also associated with premature mortality [3], higher symptoms of depression [4], and cognitive decline [5]. Nevertheless, findings from studies such as the PRISM Trial [6] have shed light on the protective role of technology adoption. Understanding digital technology adoption is critical given documentation of the benefits ranging from memory enhancements [7] to mood benefits in terms of a decrease of depressive states [8] and lower feelings of loneliness, as well as a better perception of quality of life and higher levels of social support [9].

**Figure 1:**
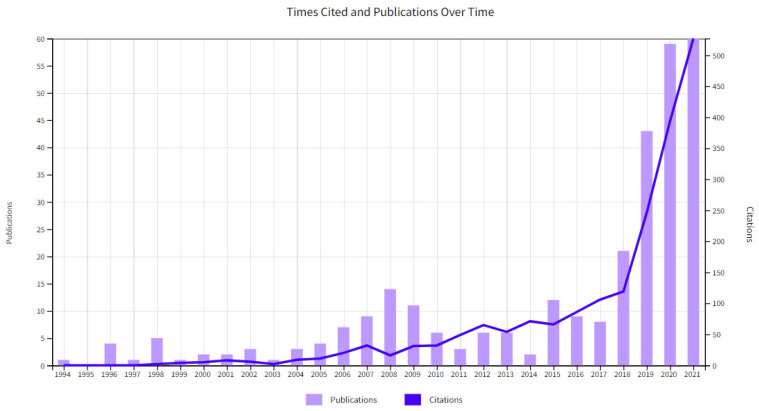
Time cited and publication over time for “*virtual assistant AND health*”.

According to the WHO in 2020, over 50 million people have dementia in the world. This number is projected to reach 82 million in 2030 and 152 in 2050. Thus, cognitive impairment is a growing concern for most countries. As is expected, this number might rise because of the effects of COVID-19 restriction measures. The unprecedented nature of the current situation has highlighted the invisibility of older people in public data analysis, where the older the person, the greater the risk of increasing their health risk and dying [10]. Unfortunately, detection of dementia in early stages is not as frequent as one might expect [11,12,13,14], being even more complex to have a diagnosis and access to care during the current pandemic. One should bear in mind that one of the main barriers to the adoption of traditional devices, such as computers, smartphones, and tablets, is related to screens, keyboards, or touch screens to enter data or commands [15]. These devices require reasonable levels of vision and manual dexterity, which can be very demanding for older people, especially those with degenerative eye and joint diseases [16]. In contrast, voice-powered smart speakers, and, particularly, virtual assistants, can avoid these limitations, as they rely on users’ speech and hearing functions. As shown in Figure 1, the literature has grown remarkably in interest in the use of virtual assistants within the healthcare field. However, age differences may occur, and more specifically, specific populations such as older adults may not be benefiting from these items.

Amazon Echo, publicly launched in 2016, is the first ever voice-controlled smart speaker powered by *Alexa*. The use of this gadget can help in the daily life of a visually impaired elderly person. With an internet connection, its users can listen to news, music, radio channels, and audio books. They can also check the time, set timers and alarms, organize personal calendars, search for data, and shop online, all with voice commands alone. With other smart home devices installed, they can control other linked systems, all without the need for physical contact. For the first time, a visually impaired person with no previous experience with computers can use these high-tech devices simply by speaking to them. Some literature has addressed systematic reviews of embodied conversational agents for patients with dementia [17], showing promising results on accessibility and acceptance of assistive technologies. However, can virtual assistants act as cognitive tests for the detection of early cognitive impairment? This question might shed light on the role of language as a biomarker of cognitive impairment. Thus, this study aimed to develop a systematic review on the development of digital devices, and specifically virtual assistants, for the detection of cognitive impairment in the older adults.

## 2. Materials and Methods

This study followed the research question guidelines of the PRISMA Statement in WOS and PubMed. General search terms with the controlled descriptors for each database were used, employing the Health Sciences Descriptors (DeCS), the Medical Subject Heading (MeSH) from MEDLINE/PubMed terms, and the descriptors and terms published in the literature. To connect these terms, we used the Boolean terms “AND” and “OR” in order to expand and restrict the search spectrum. The specific descriptors were:

*virtual assistant OR digital assistant.*

*cognitive impairment OR cognitive dysfunction OR cognitively impaired OR dementia.*

**Figure 2:**
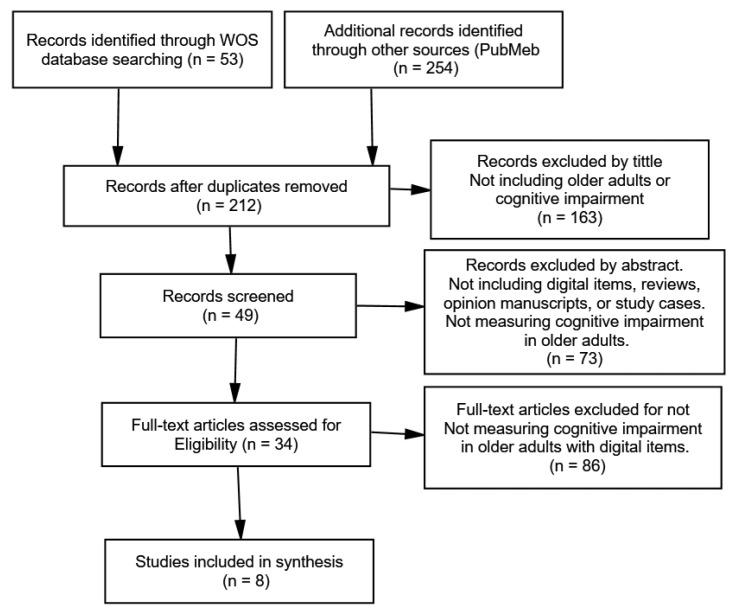
Prisma flow chart on decisions carried out.

Grey literature was addressed through some bibliographic databases include dissertations/theses, such as CINAHL and PsycINFO, but the number of results was equal to 0 and were not included in the Prima Flowchart. For data extraction, an Excel worksheet was created (Microsoft Office^®^, Microsoft, Redmond, DC, USA) based on the recommendations of the “Cochrane Handbook for Systematic Reviews”. The study selection was performed in two stages by two independent reviewers: (a) first, reading the titles and abstracts and article selection by at least one of the reviewers individually, and (b) second, reading the full-text and article selection by both reviewers in agreement; when disagreement was present, a third reviewer was consulted. The articles that were eliminated at this stage for not meeting the inclusion criteria had the reason for their exclusion described in Figure 2. Data extraction included the following information: (a) general information about the study (i.e., title, year and period of publication, first author, and country of origin); (b) information about the methodology; (c) information about the sample (i.e., sample selection and collection method, sample size, age, and gender distribution); and (d) information about the outcome (mild cognitive impairment). The inclusion criteria were (a) to be an observational study (i.e., cross-sectional, cohort, or case-control studies) in older adult subjects (>60 years of age), (b) to examine cognitive impairment, and (c) to be published in English. The exclusion criteria involved (a) studies in which the disease or outcome was not cognitive impairment and (b) studies not involving a digital item for cognitive impairment detection.

## 3. Results

First, Prisma flow chart on the decision made is offered in Figure 2. The main results can be described in Table 1. Two portals were thus used to search for data. After the screening process, only eight papers were eligible, as described in Table 1.

**Figure 3:**
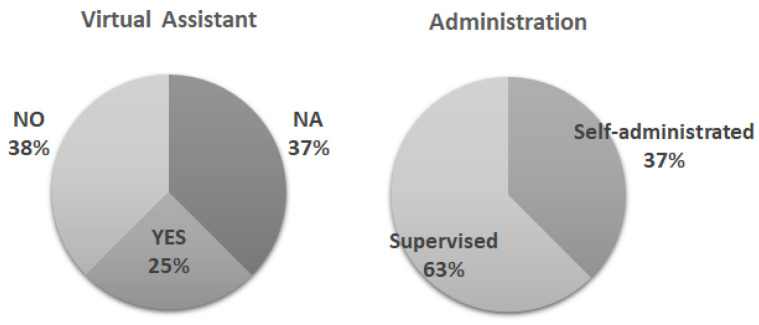
Virtual assistant use and type of administration.

Many of these solutions provide information related to the main screening test in the field of cognitive impairment. More precisely, these results involve the following areas for most cases: memory, attention, executive function, processing speed, and visuospatial function. While adaptations of common pencil and paper-based tests such as the MMSE can be found for telephone assessment, to our knowledge, this has not been automated with AI, although the studies in Table 1 show promising results along these lines. For this reason, as depicted in Figure 3, the presence of a virtual assistant was examined as a main outcome of interest.

Of note, only two studies employed this tool, while the other three did not provide enough information to examine this issue. With regards to the type of administration, the majority employed a supervised mode, and only three studies had a self-administration procedure that was monitored. This could be related to participants’ characteristics, as some studies did include participants with dementia, but others did not and excluded these participants in their criteria [18,21,22,25]. Moreover, two of the studies included not only older people but also adults [24,25]. While in the eMoca study, an age range of participants from 20–61 years old were included, in STAC, participants from 18 to 85 years old were included. Although it could be considered a conflict with the inclusion criterion of being over 60 years of age in the present study, they were included by partially covering this information. In sum, the most frequent solution was the type of task developed on the iPad with a short time frame.

## 4. Conclusions and Discussion

This study aimed to examine the development of virtual assistants and digital technology in the assessment of cognitive decline in older adults. The main results can be summarized as follows: (i) the number of results of digital devices to measure cognition in older adults is small and most do not benefit from virtual assistants, (ii) most devices are administered in a supervised manner, (iii) tablet use is the most popular type of device for this purpose, and (iv) administration times are usually short, mostly not exceeding 30 min.

Based on the current findings, literature in this field seems to be in progress. This could be considered as surprising, given the advance of virtual assistants, as well as the interest of different companies in the field. The main explanation would be related to the barriers that older people encounter to access to technology. This hypothesis would be supported by the large number of devices monitored in the review. Considering that cognitive impairment likely begins many years, at most decades, before the onset of clear symptoms [13], an opportunity for prevention though a virtual assistant might make it possible to make a diagnosis faster and, moreover, to examine language biomarkers before symptom onset [14]. In this way, we must also consider that the field of machine learning is relatively new compared to other fields. As language can be understood as a first sign of cognitive decline, analysis in this area is a promising line of research based on NLP. The interest in computerized analysis of spoken language through natural language processing (NLP) and machine learning techniques has grown in the last decade, and so has the availability of numerous algorithms for analysis and classification of language production [26].

The question of self-administration raised in this research is of relevance. By no means can these replace professionals, but they can provide them with a large amount of information in a systematised way thanks to AI and the learning process of algorithms. However, older adults have often not been considered as beneficiaries of these technological advances, given a certain reluctance in adopting digital technology, as described in previous literature [15]. Even if the most relevant result of this study is the low number of devices for measuring cognitive impairment in the elderly, the use of iPad-like tools is also striking. This could be due to a better visual presentation of the results under a bigger screen that allows manual manipulation. If we compare a smartphone with an iPad, the size for manual interaction is much larger in the latter case, which could improve usability for this type of user. This could explain the higher usage as well as the preference for supervised types of data collection. Nevertheless, this is also a field of interest where a virtual assistant might be advantaged. One should bear in mind that entering data or commands can be very demanding for older people. In contrast, a virtual assistant can avoid this adoption limitation, as older people rely on users’ speech, e.g., a user saying, ‘ok Google’.

Lastly, it is expected that healthcare professionals will encounter an increasing number of technology-savvy older patients in the coming years. This approach and related technologies might have the important advantage of representing a record of natural, spontaneous language, outside the diagnostic configuration of conventional neuropsychological language testing, potentially applicable to broad segments of the population with low-cost tools. Research on this front can provide volumes of data needed in healthcare guidance for the purpose of treatment monitoring or simply optimising costs and functionality to make them more accessible and user-friendly for this population. To date, the full benefits that this technology can bring seems to remain undercover. In the health area, verbal fluency is employed as a proxy measure of executive functioning, as it has been linked to working memory, inhibitory control, and cognitive flexibility and even been shown to be closely linked to fluid intelligence, among others [27], e.g., verbal fluency involves the need to remember what has already been said (thus preventing perseveration) and the need to maintain the task goal in mind while generating subgoals and exemplars. On a theoretical level, several theories have tried to explain the disproportionate effect of aging on semantic fluency compared to letter fluency, most of them related to executive functioning and to speed of processing. Therefore, if virtual assistants can measure language, it seems imperative to take advantage of this technology in the elderly. Specifically, language analysis in clinical contexts could quantify many aspects of language, both at the segmental and suprasegmental levels, such as prosody and rhythm, that are not explored by conventional language tests and cognitive impairment screenings for older adults [28]. Moreover, it should be noted that results from word processor analysis have shed light on changes such as writing deficits described as markers for dementia [29]. Thus, an opportunity for prevention providing an early diagnosis based on language [14] and the use of virtual assistants that involve NLP might emerge. Furthermore, verbal fluency is a task that is predictive of incident cognitive impairment [30].

## Figures and Tables

**Table 1:** Studies included for analysis. Country, tools, and sample description.

Authors	Country	Acronym	Tool	Sample	Mean Age	Duration
Gorodeski, et al. (2019) [18]	United States	PST	iOS application	60	69	4.75 min
Kang et al. (2018) [19]	South Korea	CoSAS-S	Tablet-based	36	68	10–15 min
Lunardini et al., 2020 [20]	Italy	TMT adaptation	web app	83	77.6	About 2 h after the paper-based tests
Maguire et al., 2019 [21]	United States	CNAD-SAGE	iPadOS	42	60.6	NA
Makizato et al. (2013) [22]	United States	NCGG-FAT	iPadOS	20	65–81	20–30
Onoda et al., 2013 [23]	Japan	CADi	iPadOS	222	71.73	10 min
Snowdon et al. (2015) [24]	Canada	eMOCA	a tablet-based	182	51.7	15.45
Wallace et al. (2017) [25]	United States	STAC	iPadOS	88	49.18	15–30

PST: Processing Speed Test; CoSAS-S: Computer Cognitive Senior Assessment System-Screen; TMT: Trail Making Test; CNAD-SAGE: computerized neuropsychological assessment devices; NCGGT-FAT: Geriatrics and Gerontology functional assessment tool; CADi: Cognitive Assessment for Dementia, iPad; eMOCA: electronic Montreal Cognitive Assessment; STAC: Touchscreen Tablet-Based Cognitive Assessment.

## Data Availability

Not applicable.
